# Supplementary material for: Isolation of an antimicrobial compound produced by bacteria associated with reef-building corals
Source: PeerJ. 2016 Aug 18;4:e2275. doi: 10.7717/peerj.2275 (PMC4994080; doi:10.7717/peerj.2275)
Supplement: Supplemental Information 1 [file peerj-04-2275-s001.docx]

| Species | Temperature | Clear zone (mm) |
| --- | --- | --- |
| *Vibrio owensii* | 28 | 5 |
| *Vibrio owensii* | 28 | 4 |
| *Vibrio owensii* | 28 | 4 |
| *Vibrio owensii* | 28 | 4.5 |
| *Vibrio owensii* | 28 | 5 |
| *Vibrio owensii* | 28 | 5.5 |
| *Vibrio owensii* | 28 | 4 |
| *Vibrio owensii* | 28 | 4.5 |
| *Vibrio owensii* | 28 | 5 |
| *Vibrio owensii* | 28 | 4 |
| *Vibrio owensii* | 28 | 4.5 |
| *Vibrio owensii* | 28 | 5.5 |
| *Vibrio owensii* | 28 | 5 |
| *Vibrio owensii* | 28 | 4 |
| *Vibrio owensii* | 28 | 4.5 |
| *Vibrio owensii* | 28 | 5 |
| *Vibrio owensii* | 28 | 4 |
| *Vibrio owensii* | 28 | 4.5 |
| *Vibrio owensii* | 28 | 4.5 |
| *Vibrio owensii* | 28 | 4 |
| *Vibrio owensii* | 32 | 0.5 |
| *Vibrio owensii* | 32 | 0.5 |
| *Vibrio owensii* | 32 | 0.5 |
| *Vibrio owensii* | 32 | 1 |
| *Vibrio owensii* | 32 | 0.5 |
| *Vibrio owensii* | 32 | 1 |
| *Vibrio owensii* | 32 | 0.5 |
| *Vibrio owensii* | 32 | 0.5 |
| *Vibrio owensii* | 32 | 1 |
| *Vibrio owensii* | 32 | 0.5 |
| *Vibrio owensii* | 32 | 0.5 |
| *Vibrio owensii* | 32 | 1 |
| *Vibrio owensii* | 32 | 0.5 |
| *Vibrio owensii* | 32 | 1 |
| *Vibrio owensii* | 32 | 0.5 |
| *Vibrio owensii* | 32 | 1 |
| *Vibrio owensii* | 32 | 0.5 |
| *Vibrio owensii* | 32 | 0.5 |
| *Vibrio owensii* | 32 | 1 |
| *Vibrio owensii* | 32 | 0.5 |
| *Vibrio coralliilyticus* | 28 | 2 |
| *Vibrio coralliilyticus* | 28 | 2 |
| *Vibrio coralliilyticus* | 28 | 1.5 |
| *Vibrio coralliilyticus* | 28 | 1 |
| *Vibrio coralliilyticus* | 28 | 2 |
| *Vibrio coralliilyticus* | 28 | 2 |
| *Vibrio coralliilyticus* | 28 | 1 |
| *Vibrio coralliilyticus* | 28 | 2 |
| *Vibrio coralliilyticus* | 28 | 2 |
| *Vibrio coralliilyticus* | 28 | 1.5 |
| *Vibrio coralliilyticus* | 28 | 2 |
| *Vibrio coralliilyticus* | 28 | 1 |
| *Vibrio coralliilyticus* | 28 | 2 |
| *Vibrio coralliilyticus* | 28 | 2 |
| *Vibrio coralliilyticus* | 28 | 2 |
| *Vibrio coralliilyticus* | 28 | 1.5 |
| *Vibrio coralliilyticus* | 28 | 1 |
| *Vibrio coralliilyticus* | 28 | 2 |
| *Vibrio coralliilyticus* | 28 | 2 |
| *Vibrio coralliilyticus* | 28 | 2 |
| *Vibrio coralliilyticus* | 32 | 0.5 |
| *Vibrio coralliilyticus* | 32 | 0.5 |
| *Vibrio coralliilyticus* | 32 | 1 |
| *Vibrio coralliilyticus* | 32 | 0.5 |
| *Vibrio coralliilyticus* | 32 | 0.5 |
| *Vibrio coralliilyticus* | 32 | 0.5 |
| *Vibrio coralliilyticus* | 32 | 1 |
| *Vibrio coralliilyticus* | 32 | 0.5 |
| *Vibrio coralliilyticus* | 32 | 0.5 |
| *Vibrio coralliilyticus* | 32 | 0.5 |
| *Vibrio coralliilyticus* | 32 | 0.5 |
| *Vibrio coralliilyticus* | 32 | 1 |
| *Vibrio coralliilyticus* | 32 | 1 |
| *Vibrio coralliilyticus* | 32 | 0.5 |
| *Vibrio coralliilyticus* | 32 | 0.5 |
| *Vibrio coralliilyticus* | 32 | 0.5 |
| *Vibrio coralliilyticus* | 32 | 0.5 |
| *Vibrio coralliilyticus* | 32 | 0.5 |
| *Vibrio coralliilyticus* | 32 | 1 |
| *Vibrio coralliilyticus* | 32 | 0.5 |
| TDA *Vibrio owensii* | 28 | 6 |
| TDA *Vibrio owensii* | 28 | 6.5 |
| TDA *Vibrio owensii* | 28 | 6 |
| TDA *Vibrio owensii* | 28 | 6 |
| TDA *Vibrio owensii* | 28 | 6.5 |
| TDA *Vibrio owensii* | 28 | 6 |
| TDA *Vibrio owensii* | 28 | 6.5 |
| TDA *Vibrio owensii* | 28 | 6.5 |
| TDA *Vibrio owensii* | 28 | 6 |
| TDA *Vibrio owensii* | 28 | 6 |
| TDA *Vibrio owensii* | 28 | 6 |
| TDA *Vibrio owensii* | 28 | 6.5 |
| TDA *Vibrio owensii* | 28 | 6 |
| TDA *Vibrio owensii* | 28 | 6.5 |
| TDA *Vibrio owensii* | 28 | 6.5 |
| TDA *Vibrio owensii* | 28 | 6 |
| TDA *Vibrio owensii* | 28 | 6 |
| TDA *Vibrio owensii* | 28 | 6.5 |
| TDA *Vibrio owensii* | 28 | 6 |
| TDA *Vibrio owensii* | 28 | 6 |
| TDA *Vibrio owensii* | 32 | 6.5 |
| TDA *Vibrio owensii* | 32 | 6 |
| TDA *Vibrio owensii* | 32 | 6.5 |
| TDA *Vibrio owensii* | 32 | 6.5 |
| TDA *Vibrio owensii* | 32 | 6 |
| TDA *Vibrio owensii* | 32 | 6 |
| TDA *Vibrio owensii* | 32 | 6.5 |
| TDA *Vibrio owensii* | 32 | 6 |
| TDA *Vibrio owensii* | 32 | 6 |
| TDA *Vibrio owensii* | 32 | 6.5 |
| TDA *Vibrio owensii* | 32 | 6.5 |
| TDA *Vibrio owensii* | 32 | 6.5 |
| TDA *Vibrio owensii* | 32 | 6.5 |
| TDA *Vibrio owensii* | 32 | 6 |
| TDA *Vibrio owensii* | 32 | 6 |
| TDA *Vibrio owensii* | 32 | 6.5 |
| TDA *Vibrio owensii* | 32 | 6.5 |
| TDA *Vibrio owensii* | 32 | 6 |
| TDA *Vibrio owensii* | 32 | 6.5 |
| TDA *Vibrio owensii* | 32 | 6 |
| TDA *Vibrio coralliilyticus* | 28 | 5 |
| TDA *Vibrio coralliilyticus* | 28 | 5 |
| TDA *Vibrio coralliilyticus* | 28 | 5.5 |
| TDA *Vibrio coralliilyticus* | 28 | 5 |
| TDA *Vibrio coralliilyticus* | 28 | 5.5 |
| TDA *Vibrio coralliilyticus* | 28 | 5.5 |
| TDA *Vibrio coralliilyticus* | 28 | 5.5 |
| TDA *Vibrio coralliilyticus* | 28 | 5 |
| TDA *Vibrio coralliilyticus* | 28 | 5.5 |
| TDA *Vibrio coralliilyticus* | 28 | 5.5 |
| TDA *Vibrio coralliilyticus* | 28 | 5 |
| TDA *Vibrio coralliilyticus* | 28 | 5.5 |
| TDA *Vibrio coralliilyticus* | 28 | 5.5 |
| TDA *Vibrio coralliilyticus* | 28 | 5.5 |
| TDA *Vibrio coralliilyticus* | 28 | 5.5 |
| TDA *Vibrio coralliilyticus* | 28 | 5.5 |
| TDA *Vibrio coralliilyticus* | 28 | 5 |
| TDA *Vibrio coralliilyticus* | 28 | 5.5 |
| TDA *Vibrio coralliilyticus* | 28 | 5 |
| TDA *Vibrio coralliilyticus* | 28 | 5.5 |
| TDA *Vibrio coralliilyticus* | 32 | 5.5 |
| TDA *Vibrio coralliilyticus* | 32 | 5.5 |
| TDA *Vibrio coralliilyticus* | 32 | 5.5 |
| TDA *Vibrio coralliilyticus* | 32 | 5 |
| TDA *Vibrio coralliilyticus* | 32 | 5 |
| TDA *Vibrio coralliilyticus* | 32 | 5.5 |
| TDA *Vibrio coralliilyticus* | 32 | 5.5 |
| TDA *Vibrio coralliilyticus* | 32 | 5.5 |
| TDA *Vibrio coralliilyticus* | 32 | 5 |
| TDA *Vibrio coralliilyticus* | 32 | 5.5 |
| TDA *Vibrio coralliilyticus* | 32 | 5.5 |
| TDA *Vibrio coralliilyticus* | 32 | 5 |
| TDA *Vibrio coralliilyticus* | 32 | 5 |
| TDA *Vibrio coralliilyticus* | 32 | 5.5 |
| TDA *Vibrio coralliilyticus* | 32 | 5 |
| TDA *Vibrio coralliilyticus* | 32 | 5.5 |
| TDA *Vibrio coralliilyticus* | 32 | 5 |
| TDA *Vibrio coralliilyticus* | 32 | 5.5 |
| TDA *Vibrio coralliilyticus* | 32 | 5 |
| TDA *Vibrio coralliilyticus* | 32 | 5 |
